# Galanin Reduces Myocardial Ischemia/Reperfusion Injury in Rats with Streptozotocin Diabetes

**DOI:** 10.32607/actanaturae.27506

**Published:** 2025

**Authors:** I. M. Studneva, L. I. Serebryakova, O. M. Veselova, I. V. Dobrokhotov, M. E. Palkeeva, D. V. Avdeev, A. S. Molokoedov, M. V. Sidorova, O. I. Pisarenko

**Affiliations:** Chazov National Medical Research Center of Cardiology, Moscow 121552 Russian Federation

**Keywords:** galanin, rat, streptozotocin diabetes, myocardial ischemia and reperfusion, mitochondrial dysfunction, myocardial energy state, cell membrane damage

## Abstract

Most clinical studies confirm the negative impact diabetes mellitus (DM) has on the course and outcome of cardiovascular complications caused by a myocardial ischemia–reperfusion injury (IRI). In this regard, the search for new approaches to IRI treatment in diabetic myocardium is of undeniable value. The aim of this work was to study the effect of galanin (G) on the size of myocardial infarct (MI), on mitochondrial functions, and on the energy state in the area at risk (AAR) in rats with type 1 diabetes mellitus (DM1) subjected to regional myocardial ischemia and reperfusion. Rat G was obtained by solid-phase synthesis using the Fmoc strategy and purified by HPLC. DM1 was induced by streptozotocin administration. Myocardial IRI was modeled by occlusion of the left anterior descending coronary artery and subsequent reperfusion. G at a dose of 1 mg/kg was administered intravenously before reperfusion. G decreased MI size and plasma creatine kinase MB (CK–MB) activity in DM rats by 40 and 28%, respectively. G injection improved mitochondrial respiration in saponin-skinned fibers in the AAR: namely, the maximal ADP-stimulated state 3, respiratory control, and the functional relationship between the mitochondrial CK–MB and oxidative phosphorylation. G provided significantly higher ATP levels, total adenine nucleotide pool, and adenylate energy charge of cardiomyocytes. It also reduced total creatine loss in myocardial AAR in DM rats. The results suggest there is a possibility of therapeutic use of G in myocardial IRI complicated by DM1.

## INTRODUCTION

If we consider the mutually reinforcing negative impact of diabetes mellitus (DM) and myocardial ischemia as two common pathologies on prognosis and a patient’s life quality, this comorbidity presents one of the most vexing challenges in modern experimental and clinical cardiology. DM patients are more likely to suffer from coronary artery occlusions, and their myocardium is more prone to ischemia–reperfusion injury (IRI) compared to non-DM individuals [1]. As a rule, cardioprotection from IRI is ineffective in DM [2]. This has to do with the defects in the PI3K/Akt and JAK2/STAT3 signaling cascades, which play the key role in cardioprotection [3]. Diabetic hyperglycemia can cause mitochondrial dysfunction by increasing the expression of the dynamin-1-like protein [4], inhibiting mitochondrial ATP-dependent K+ channels [5], and inactivating hypoxia-inducible factor 1α (HIF-1α) [6]. These metabolic changes contribute to the desensitization of the diabetic myocardium to therapeutic interventions against IRI. In this regard, the search for new pharmacological targets for preventing and treating myocardial IRI in DM is of undeniable value.

An important role in cardiovascular regulation in diseases has recently been attributed to the galaninergic system [7]. The neuropeptide galanin (G; GWTLNSAGYLLGPHAIDNHRSFSDKHGLT-NH_2_) is widely found in the central and peripheral nervous systems and other tissues [8]. In peripheral organs, including the heart, G acts not only through neuronal mechanisms, but it also activates the galanin receptor GalR1-3 [9]. We recently showed that intravenous injection of G to rats after regional myocardial ischemia significantly reduced cardiomyocyte necrosis [10]. This effect was mediated by GalR2 activation and significantly reduced in the presence of M871, a GalR2 antagonist [11]. Reduced myocardial infarction (MI) in the presence of G was accompanied by a decreased formation of the hydroxyl radical adduct 5,5-dimethyl- pyrroline-N-oxide-OH and lipid peroxidation products (LPP) in the area at risk (AAR) upon blood flow restoration. G can also inhibit the free radical oxidation of low-density lipoproteins in human plasma [12]. It is important to note that G prevented hyperglycemia in streptozotocin (STZ)-induced DM in rats, improved the metabolic state of DM animals thanks to an increase in the mitochondrial respiratory function, and reduced LPP formation in plasma [13]. We hypothesized that this peptide, which improves energy production in cardiac mitochondria and reduces oxidative stress, is a promising agent for reducing IRI in type 1 DM (DM1). The G effect on ischemic myocardium exposed to DM has never been studied before. To test this hypothesis, we used G during the reperfusion period after regional myocardial ischemia in rats with STZ-induced hyperglycemia. We used the following cardiac damage criteria: MI size and plasma activity of the necrosis markers creatine kinase-MB (CK–MB) and lactate dehydrogenase (LDH). To understand the mechanisms of G action, we placed the main focus on the energy state in the AAR and mitochondrial function, which was characterized by respiration in saponin-skinned myocardial fibers.

## EXPERIMENTAL

**Reagents**

Fmoc-protected amino acid derivatives were purchased from Novabiochem and Bachem (Switzerland); reagents for peptide synthesis were obtained from Fluka Chemie GmbH (Switzerland). Enzymes and the chemicals used to determine metabolites and evaluate myocardial fiber respiration were purchased from Merck Life Science LLC (Russia). Solutions were prepared using deionized water (Millipore Corp., USA).

**Peptide G synthesis and chromatography**

Peptide G was obtained by convergent solid-phase synthesis through condensation of peptide segments, which, in turn, were obtained either on a polymer surface or in solution. Peptide G was purified by preparative HPLC to 98% purity on a Knauer chromatograph (Germany) using a Kromasil 100-10 ODS column (Sweden) (30 × 250 mm) [14]. Analytical HPLC was performed on a Kromasil 100-5 C18 column (4.6 × 250 mm) with a 5-μm sorbent particle size. The following eluents were used: 0.1% TFA as buffer A and 80% acetonitrile in buffer A as buffer B. The elution was carried out in a linear buffer B gradient ranging from 20 to 80% for 30 min at a rate of 1 ml/min. Detection was performed at λ = 220 nm (Supplementary materials; *Fig. S1*). The peptide structure was confirmed by MALDI-TOF/TOF mass spectrometry on an UltrafleXtreme Bruker Daltonics GmbH mass spectrometer (Germany) equipped with a UV laser (Nd) (Supplementary materials, *Fig. S2*). Peptide G characteristics are provided in *Table 1*.

**Table 1:** Characteristics of peptide G

Amino acid sequence	Molecular weight, g/mol	MALDI-TOF, m/z	Solubility in water, mg/ml	Purity, HPLC, %
GWTLNSAGYLLGPHAIDNHRSFSDKHGLT-NH_2_	3164.45	3163.474 [M + H]^+^	> 40	98.10

**Experimental design**

**Fig. 1:**
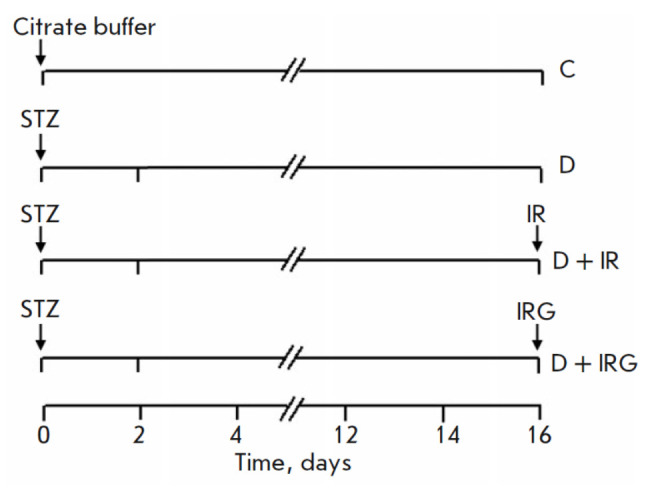
Experimental protocol scheme. D – rats receiving STZ (60 mg/kg in 0.1 M citrate buffer; pH 4.5; intravenously); D + IR – DM rats (STZ, 60 mg/kg; intravenously) subjected to regional myocardial IRI; D + IRG – DM rats (STZ, 60 mg/kg; intravenously) subjected to regional myocardial IRI, receiving G (1 mg/kg, bolus intravenous administration at the onset of reperfusion). Cit. buffer – 0.1 M citrate buffer (pH 4.5); STZ – streptozotocin

Male Wistar rats weighing 280–290 g were used in the study. The animals were procured from the Stolbovaya Animal Nursery of the Scientific Center for Biomedical Technologies (Moscow, Russia). The rats were kept in individual cages at 20–25°C in the natural light–dark cycle with free access to a standard pelleted diet and water. All the animals were weighed prior to the study. After a 24-h fasting period, the rats were taken in for blood collection. Blood was collected from the tail vein of 10 rats to determine the glucose plasma level and CK–MB and LDH activities in the animal plasma. The rats were then anesthetized with 2,2,2-tribromoethanol (avertin, 1 mg/kg intraperitoneally; Merck, Russia); their hearts were excised to assess energy metabolism parameters (*n *= 5) and mitochondrial respiration parameters in left ventricular (LV) fibers (*n *= 5) (initial state group; IS). The remaining animals were randomly divided into 5 groups of 15 rats, each: control (C), cardiac IRI (IR); diabetes mellitus (D), diabetes mellitus followed by cardiac IRI (D + IR), and diabetes mellitus with cardiac IRI and G administration at the onset of reperfusion (D + IRG). In the IR group, cardiac injury was modeled by occlusion of the left anterior descending coronary artery (LAD), followed by reperfusion [11]. Acute DM1 was induced by a single STZ injection (60 mg/kg intravenously) [15]. DM was confirmed based on an increase in the blood glucose level to ≥12 mM two days after STZ injection. The glucose level did not decrease during 16 days of experiment in all STZ-receiving animals. The D + IR group received a single STZ injection (60 mg/kg intravenously). After a 16-day experiment, the D + IR animals were subjected to LAD occlusion and reperfusion for the same period of time as the IR group. The D + IRG group received a single STZ administration (60 mg/kg intravenously); IRI was modeled after 16 days. Peptide G in saline was administered intravenously at a bolus dose of 1 mg/kg at the beginning of reperfusion. The G dose was selected based on our previous results [11]. The control rats received a single intravenous injection of 0.1 M citrate buffer (pH 4.5; STZ solvent). Body weight and the blood glucose level in the experimental animals were determined on a weekly basis. After a 16-day study, blood samples were collected from the tail vein of the rats of all groups to determine the plasma activities of CK–MB and LDH. The AAR and MI sizes were assessed in the hearts of five animals from the IR, D + IR, and D + IRG groups by histochemical analysis. The hearts of five rats from the experimental groups were isolated after anesthesia with avertin (1 mg/k intraperitoneally) and frozen in liquid nitrogen using Wollenberger forceps for subsequent metabolite analysis. The remaining five animals from the same groups were used to evaluate the mitochondrial respiration parameters in LV fibers. The experimental protocol is represented schematically in *Fig. 1*.

**Rat model of regional myocardial ischemia and reperfusion**

The IR, D + IR, and D + IRG groups of animals were anesthetized with 20% urethane (1 200 mg/kg of body weight, intraperitoneally) and artificially ventilated with room air through thoracotomy using the KTR-5 system (Hugo Sacks Electronik, Germany). Mean arterial pressure and heart rate were monitored. Parameters were recorded during the experiment using a USB-6210 analog-to-digital converter (National Instruments, USA) and the LabView 7 system software (National Instruments). The preparation period was followed by a period of hemodynamic parameter stabilization (30 min). The animals were then subjected to 40-min LAD occlusion, followed by 60-min reperfusion. In the D + IRG group, simultaneously with the beginning of reperfusion, peptide G was injected intravenously at a bolus dose of 1.0 mg/kg body weight. In the IR and D + IR groups, the same volume of a physiological solution was administered intravenously as a bolus after a period of regional ischemia. At the end of the experiment, the LAD was reoccluded and 2 ml of a 2% Evans solution were injected into the jugular vein to determine the AAR and the intact myocardial region. The heart was then excised, and LV was isolated to determine the MI size.

**Determination of the MI size**

The frozen LV was incised perpendicular to the long cardiac axis into 4- to 5- ~1.5- to 2.0-mm-thick sections. The sections were incubated for 10 min in a 1% solution of 2,3,5-triphenyltetrazolium chloride (TTC) in 0.1 M potassium phosphate buffer (pH 7.4 at 37°C). The resulting samples were scanned; MI and AAR were determined by computer planimetry using the ImageJ software (NIH, USA). Sections were then weighed to determine the LV mass. The AAR/LV and MI/AAR ratios were calculated and expressed in % for each group [11].

**Assessment of cardiomyocyte membrane damage**

Damage to cardiomyocyte membranes was assessed based on the increase in plasma LDH and CK–MB activities. Approximately 0.5 ml of blood was collected into heparin tubes from a venous catheter at the baseline (prior to LAD occlusion) and 1 h after reperfusion. Enzyme activity was evaluated in plasma using BioSystems kits on a Shimadzu UV-1800 spectrophotometer (Japan) at λ = 340 nm.

**Respiration in permeabilized myocardial fibers**

Saponin-permeabilized fibers from rat LV were prepared using a modified approach [16]. LV fiber respiration parameters were assessed using complex I substrates: 10 mM glutamate and 5 mM malate. An Oxygraph plus system (HansaTech Instruments, UK) was used for analysis. The resulting values were expressed as nmol O_2_/min/mg dry weight. The respiration rate in state 3 (V3) was achieved by adding 2 mM ADP. Fiber dry weight was determined after overnight drying at 95°C. The respiration parameters of each LV fiber sample were measured twice. The respiration rate in state 2 (V2) was evaluated base on the oxygen consumption rate after the addition of 10 mM glutamate and 5 mM malate in the absence of ADP. Mitochondrial function was assessed based on a measurement of the respiratory control (RC) value, which was calculated as the V3/V2 ratio. The integrity of the outer mitochondrial membrane was determined by adding 10 μM cytochrome *c *after maximal respiration stimulation using 2 mM ADP; the obtained values were expressed as the Vcyt c/VADP ratio in %. The degree of functional coupling between mitochondrial creatine kinase (mt-CK) and oxidative phosphorylation (OP) was assessed by adding 30 mM Cr to fibers in the presence of ADP at a submaximal concentration (0.1 mM) and calculated as the (VCr – VADP)/VADP ratio (%) [17].

**Assessment of metabolite content in the AAR**

After reperfusion, the AAR was quickly isolated from the LV and frozen using a Wollenberger clamp cooled in liquid nitrogen. The frozen tissue was homogenized in cold 6% HClO_4_ (10 ml/g of tissue) in an Ultra-Turrax T-25 homogenizer (IKA-Labortechnik, Germany). Proteins were precipitated by centrifugation (Sorvall RT1 centrifuge, Thermo Fisher Scientific, USA) at 2 800 *g *at 4°C for 10 min. Supernatants were neutralized with 5M K_2_CO_3_ to pH 7.4. The KClO4 precipitate was separated by centrifugation under the same conditions. Protein-free extracts were stored at –70°C prior to metabolite determination. The dry weight of homogenized tissue was determined after drying samples at 110°C for 24 h. The ATP, ADP, AMP, PCr, and Cr levels in tissue extracts were determined by modified enzymatic methods [18] using a Shimadzu UV-1800 spectrophotometer (Japan).

**Statistical analysis**

The SigmaPlot 11.2 software package (SysStat, USA) was used for the statistical analysis. Values are presented as a mean ± standard error of the mean (M ± m). Differences between the groups were statistically confirmed using the analysis of variance (ANOVA). Student’s t-test with Bonferroni correction was used to compare several groups with the control. Differences were considered statistically significant at* p * < 0.05.

## RESULTS

**Body weight and the blood glucose level**

At baseline (day 1 of the experiment), animal weight did not differ significantly between the groups (*Table 2*). A progressive increase in body weight was noted during the observation period in the control group. In the diabetic group, no weight gain was observed until day 9 of the study (one week after an increase in the blood glucose level higher than 12 mM in the presence of STZ). At the end of the study, the body weight in this group was, on average, 11.8 and 22.1% lower than that in the initial state and in the control (*p* < 0.02 and *p* < 0.001, respectively). A similar change in the body weight was noted in the D + IR and D + IRG groups. No differences in the body weight were found in DM animals on the last day prior to IR heart damage modeling and G injection.

**Table 2:** Changes in body weight and blood glucose level in the studied animal groups

Group	Body mass, g		
	Day 1	Day 9	Day 16
C	326.2 ± 11.7	344.2 ± 3.5	379.2 ± 4.5^*^
D	335.2 ± 2.5	348.7 ± 3.3^*^	295.5 ± 14.5^*$@^
IR	340.6 ± 3.6	–	–
D + IR	338.2 ± 1.7	376.5 ± 2.6^*@^	291.3 ± 4.6^*$@^
D + IRG	336.2 ± 2.3	380.0 ± 4.3^*@#^	321.2 ± 13.0^$@+^
Blood glucose level, mM			
C	6.1 ± 0.2	–	6.3 ± 0.2
D	5.3 ± 0.5	22.4 ± 0.8^*^	23.8 ± 1.7^*@^
IR	5.1 ± 0.4	–	–
D + IR	4.9 ± 0.6	26.8 ± 3.3^*^	21.3 ± 5.1^*@^
D + IRG	5.0 ± 0.2	25.0 ± 2.0^*^	21.5="" ± 3.3^*@^

Data are presented as M ± m (n = 15). p < 0.05 vs:

^*^ – value on day 1,

^$^ –="" value on day 9,

^@^ –="" control,

^#^ –="" D,

^+^ –="" D + IR.

**The effect of DM and peptide G on the size of the myocardial infarction**

**Fig. 2:**
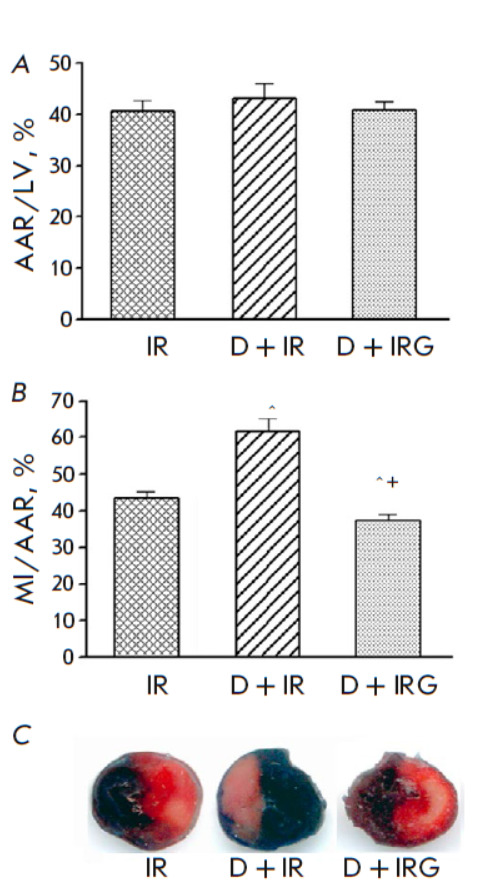
The sizes of the AAR (*A*), MI (*B*), and LV sections (*C*) stained with 2,3,5-triphenyltetrazolium chloride at the end of reperfusion in groups with regional myocardial IRI. M ± m for groups of 5 animals are shown. *p* < 0.05 compared to: ^ – IR, + – D + IR

Histochemical analysis of LV sections at the end of reperfusion revealed no differences in AAR sizes between the IR, D + IR, and D + IRG groups (*Fig. 2*). AAR/LV values were similar in these groups: 41.3 ± 1.3% on average. This means that IRI modeling was standard in all the animals. In the IR group, the MI size, expressed as the MI/AAR ratio, was 43.4 ± 1.6%. In the presence of STZ, the MI size had increased 1.4-fold compared to the IR group by the end of the experiment (*p *= 0.002). Reperfusion with G significantly decreased the MI/AAR in the DM rats: this value was 40% lower in the D + IRG animals compared to the D + IR group. *Figure 2B* shows the localization of the necrotic zone in LV sections after staining with TTC. An increase in the formation of red formazan crystals due to TTC reduction by NAD^+^ and NADP^+^-dependent dehydrogenases in the D + IRG group indicates a decrease in the MI intensity in the presence of G.

**Plasma activities of CK–MB and LDH**

**Fig. 3:**
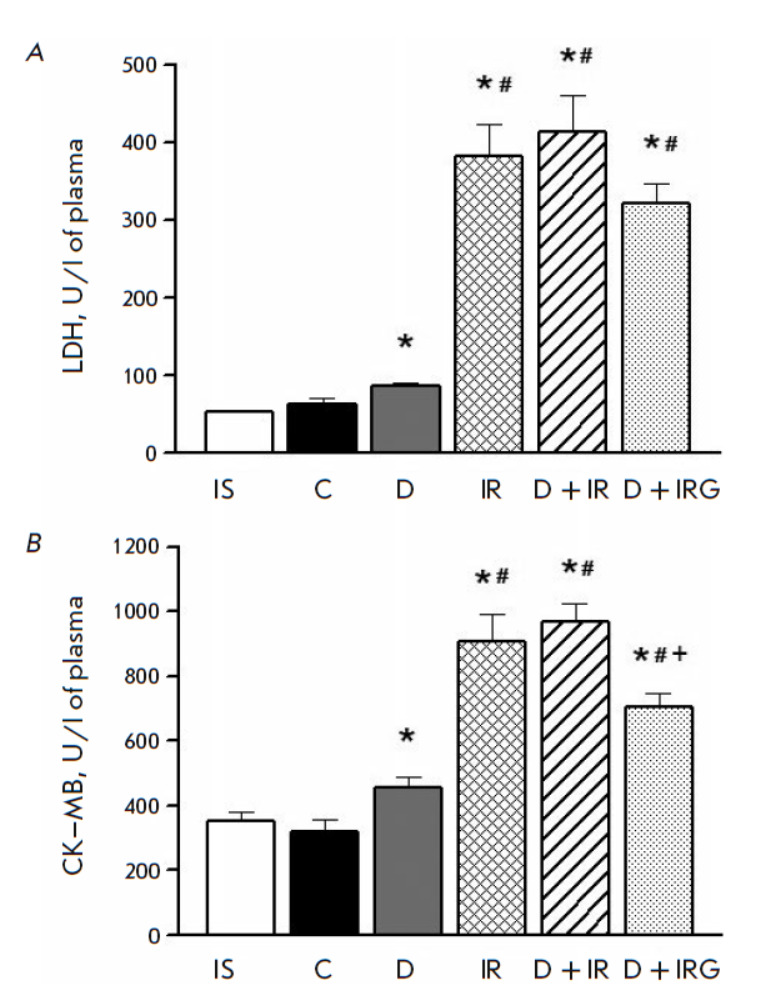
Lactate dehydrogenase (LDH, *A*) and creatine kinase-MB (CK-MB, *B*) activities in rat plasma. Values represent M±m for groups of 5 animals. *p* < 0.05 compared to: * – C, # – D, + – D + IR. IS – initial state

The CK–MB and LDH activities in the control rats did not differ from those at baseline (*Fig. 3A,B*). STZinduced DM development resulted in a significant increase in the CK–MB and LDH activities by the end of the experiment compared to the control (*p *= 0.027 and *p *= 0.046, respectively). IRI modeling had significantly increased the CK–MB and LDH activities by the end of reperfusion compared to the control (*p* < 0.001). The CK–MB and LDH values were 2.0- and 4.4-fold higher, respectively, compared to the DM group (*p* < 0.001). Regional IRI in the DM animals of the D + IR group did not cause a significant increase in the activity of necrosis markers compared to the IR group. Bolus intravenous administration of G at the onset of reperfusion reduced the CK–MB activity 1.4-fold compared to the D + IR group (*p* = 0.006). The LDH activity in the D + IR group in the presence of G decreased insignificantly compared to D + IR rats (*p *= 0.085).

**Respiration in saponin-skinned fibers**

**Fig. 4:**
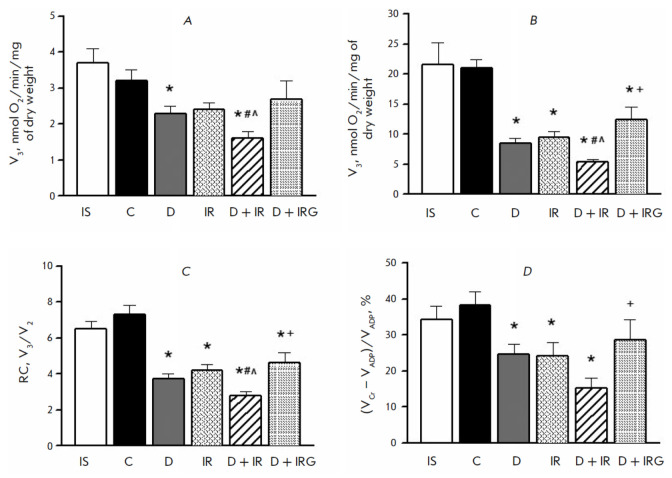
Mitochondrial respiration parameters in saponin-skinned LV fibers in the presence of 10 mM glutamate and 5 mM malate. (*A*) – oxygen consumption rate in state 2 (V2); (*B*) – oxygen consumption rate in state 3 (V3); (*C*) – respiratory control index = V3/V2; (*D*) – rate of functional coupling between mt-CK and OP (VCr – VADP)/VADP, %. Values represent M ± m for groups of 5 animals. *p* < 0.05 compared to: * – C, # – D, ^ – IR, and + – D + IR. IS – initial state

After 16 days into the experiment, no differences in the respiratory rate in states 2 and 3, the RC value, or the degree of functional relationship between mt- CK and OP were observed compared to the control group and the baseline (*Fig. 4*). A decrease in both V2 and V3 was observed in rats receiving STZ: by 28 and 60% compared to the control, respectively (*p* < 0.05 and *p* < 0.001, respectively). This resulted in a twofold decrease in RC (*p* < 0.001). The degree of mt-CK functional activity in the DM animals, evaluated in the Cr test, decreased 1.6-fold compared to the control (*p* < 0.001). Similar changes in the mitochondrial respiratory function in the AAR were caused by myocardial IRI. Average respiratory values did not differ significantly from those in the D group. The combined effect of STZ and IRI worsened respiration in states 2 and 3 compared to the DM rats (*p *= 0.038 and *p* < 0.01, respectively) and IRI animals (*p *= 0.022 and 0.004, respectively). This led to a decrease in RC compared to the groups D and IR (*p *= 0.037 and* p *= 0.05, respectively). The functional activity of mt- CK in the D + IR group was noticeably lower compared to the groups D and IR; however, the differences between the groups were statistically insignificant. Administration of peptide G to DM animals after regional myocardial ischemia increased the maximum ADP-stimulated state 3 and RC 2.3- and 1.6-fold, respectively, compared to the D + IR group (*p *= 0.011 and *p *= 0.022, respectively). The functional relationship between mt-CK and OP in the D + IRG group increased 2.4-fold compared to the D + IRG group. Representative respiratory protocols demonstrating changes in state 3 in the studied groups are presented in the Supplementary material (*Fig. S3*). The addition of 10 μM cytochrome *c *did not affect ADP-stimulated respiration in the D, D + IR, and D + IRG groups at the end of the experiment as compared to the control. The percentage ratio of Vcyt c/VADP in these groups averaged 103.5 ± 1.9%, indicating the absence of damage to the outer mitochondrial membrane in the presence of STZ and under IR conditions.

**Myocardium energy state**

**Table 3:** Energy state of rat myocardium in the studied groups

Parameter	IS	C	D	IR	D + IR	D + IRG
ATP	20.16 ± 1.27	19.16 ± 1.56	14.53 ± 1.21^*^	10.34 ± 1.45^*^	8.11 ± 0.44^*#^	11.27 ± 1.04^*+^
ADP	5.47 ± 0.43	5.36 ± 0.53	4.93 ± 0.68	4.54 ± 0.51	4.98 ± 0.27	5.69 ± 0.34
AMP	1.03 ± 0.14	1.13 ± 0.24	1.02 ± 0.27	0.97 ± 0.14	1.75 ± 0.28^^^	2.20 ± 0.15^*#^^
ΣAN	26.66 ± 1.87	25.68 ± 1.90	20.43 ± 1.24^*^	15.86 ± 1.12^*#^	14.83 ± 1.02^*#^	18.68 ± 1.35^*+^
AEC	0.85 ± 0.01	0.85 ± 0.02	0.82 ± 0.01	0.79 ± 0.02	0.71 ± 0.01^*#^^	0.75 ± 0.01^*#+^
PCr	25.34 ± 1.98	25.29 ± 1.39	15.62 ± 0.95^*^	13.86 ± 2.02^*^	17.06 ± 1.54^*^	18.89 ± 1.25^*^
Cr	37.21 ± 2.77	34.98 ± 1.36	32.54 ± 2.77	31.54 ± 2.67	30.57 ± 1.47	34.42 ± 2.41
ΣCr	62.55 ± 2.15	60.27 ± 1.37	48.16 ± 2.03^*^	45.40 ± 2.33^*^	47.63="" ± 0.74^*^	52.86="" ± 1.26^+^^

^*^Statistically significant.

Data are presented as M ± m (n = 15) and expressed for metabolites in μmol/g of dry weight.

IS – initial state.

ΣAN = ATP + ADP + AMP;

AEC = (ATP + 0.5ADP)/ΣAN;

ΣCr = PCr + Cr.

p < 0.05 vs:

^*^ – C and IS,

^#^ – D,

^^^ – IR,

^+^ – D + IR.

On day 16 of the experiment, the ATP, ADP, AMP, PCr, and Cr levels in LV in the control did not differ statistically significantly from their initial values (*Table 3*). A reliable decrease in the ATP, ΣAN, PCr, and SCr levels was noted in the DM animals compared to the controls (*p* < 0.05–0.001). Myocardial IRI had a stronger effect on ATP and ΣAN in the AAR: these parameters were decreased on average 1.3-fold compared to the controls (*p* < 0.003 and *p* < 0.002, respectively). STZ injection and subsequent regional IRI increased the loss of ATP and ΣAN in the AAR compared to the DM animals (*p *= 0.001 and *p *= 0.008, respectively). These changes in the adenine nucleotides content led to a decrease in the adenylate energy charge (AEC) of cardiomyocytes compared to the groups D and IR (*p* < 0.01 and *p* < 0.001, respectively). There were no significant changes in the PCr–Cr system in the AAR of the animals in the D + IR group compared to the groups D and IR. Administration of peptide G to DM animals at the beginning of reperfusion improved the energy state in the AAR by the end of reperfusion. This manifested itself in maintenance of higher ATP and ΣAN levels compared to the D + IR group (1.4- and 1.25-fold; *p* = 0.023 and *p *= 0.04, respectively) and a significantly higher cardiomyocyte adenylate energy charge (AEC) (*p* = 0.022). In the presence of peptide G, the ΣCr level in the AAR was higher than that in the D + IR group (*p* = 0.007) and did not differ significantly from the controls.

## DISCUSSION

In addition to hyperglycemia and a lack of body weight gain, STZ-induced DM1 modeling was accompanied by depletion of high-energy phosphate reserves and a subsequent decrease in the myocardial ΣAN and ΣCr levels in the rats. The detected impairments of the myocardial energy supply were accompanied by a slump in the maximum ADP-stimulated oxygen consumption rate in state 3 and a decrease in mt-CK functional activity, as estimated in the Cr test. These changes in mitochondrial respiration are usually associated with a lower ATP production [19] and increased ROS generation [20]. The effect of STZinduced DM was accompanied by an increase in the circulating levels of CK–MB and LDH, which indicates myocardial injury. Increased CK–MB and LDH activities in plasma were previously detected in STZinduced diabetic cardiomyopathy models in laboratory animals [21] and DM patients [22]. Subsequent myocardial IRI in STZ-receiving rats exacerbated the necrotic damage to the LV (up to 25.6%) and significantly elevated the activity of both necrosis markers in the plasma compared to the DM animals. Necrotic death of cardiomyocytes in the AAR was accompanied by a deterioration of the mitochondrial respiratory function, greater losses of ATP and ΣAN compared to the DM rats, and a decrease in cardiomyocyte AEC by the end of reperfusion. It should be noted that the combined effect of STZ and IRI significantly increased the MI size, expressed as the MI/AAR ratio (%), compared to IRI alone.

In the present work, we demonstrated for the first time the protective effect of G administration at the beginning of reperfusion after a regional ischemia period in rats with DM1. G significantly reduced the MI size and plasma CK–MB activity in these rats compared to the D + IR animals. These effects can be also due to a reduction in mitochondrial dysfunction, as indicated by an increase in ADP-stimulated respiration in state 3, RC, and an improvement in functional coupling between mt-CK with OP. This resulted in an increase in the ATP, ΣAN and AAR of cardiomyocytes and improved ΣCr preservation in the AAR. Previously, we established the ability of G to reduce the myocardial reperfusion injury in rats *in situ*, which manifested itself in MI size reduction and a decrease in damage to the cardiomyocyte membrane [23]. This was due to a decreased production of ROS and LPP in reperfused myocardium. In the present study, excessive ROS and LPP production induced by diabetic hyperglycemia and subsequent regional myocardial IRI could have been the leading cause of mitochondrial dysfunction and necrotic cell death [24]. It is possible that the protective effect of G may have to do with its antioxidant properties: increased expression of the *SOD*, *CAT*, and *GSH-Px *genes encoding enzymes of the myocardial antioxidant defense system and/or the ability to intercept ROS and inhibit LPP [12, 23].

In addition to the regulation of free radical processes, activation of various G signaling pathways upon binding to GalR1-3 receptors can contribute to a reduction in cell damage [10]. This knowledge is of fundamental importance, since DM disrupts the intracellular signaling cascades that are activated by RISK kinases. These kinases are responsible for increased cell resistance to damage, primarily the PI3K/Akt signaling pathway [2, 3]. The main factors of G-activated intracellular signaling are presented in the Supplementary material (*Fig. S4*). The most physiologically significant factors induce a stimulation of the glucose uptake by cardiomyocytes, an inhibition of the proapoptotic proteins BAD/BAX, caspase- 3, and caspase-9, inhibition of mitochondrial permeability transition pore (mPTP) opening, and an increase in the expression of peroxisome proliferatoractivated receptors (PPAR). These adaptive mechanisms play a crucial role in reducing ATP production in DM and myocardial reperfusion [25]. A decrease in cardiomyocyte apoptosis in *in vivo *models is known to be accompanied by a reduction in the MI size and an improvement in cardiac contractile function [26]. Inhibition of mPTP opening promotes cell survival and motility [27], while PPARγ expression stimulates glucose uptake and oxidation by cardiomyocytes [28]. The receptor nature of the action of G is also evidenced by the fact that GalR2 blockade with the selective antagonist M871 in myocardial IRI significantly weakens the G protective potential, increasing MI size and the plasma activity of necrosis markers [11]. It is important to note that the effect of full-length galanin, which binds to all GalR1-3 receptor subtypes, is reproduced by native and modified N-terminal G fragments, which possess a high affinity for GalR2 [10]. This suggests a potential role for GalR2 activation in the treatment and prevention of myocardial IRI in DM patients.

## CONCLUSION

**Fig. 5:**
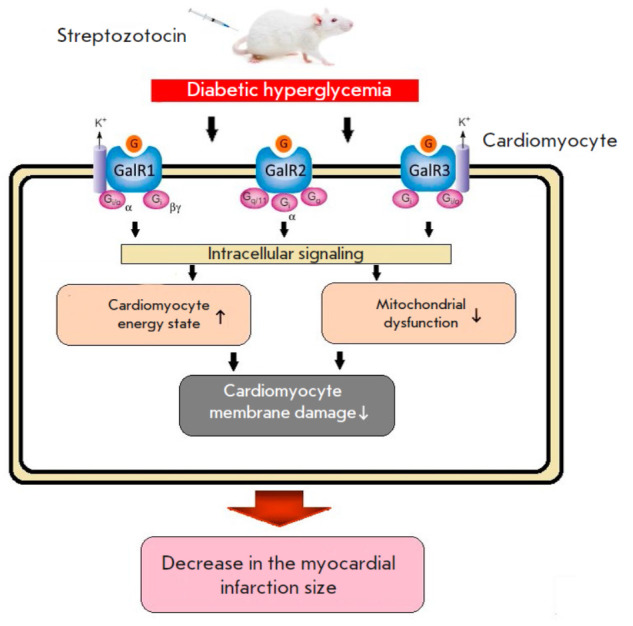
Activation of intracellular G signaling during streptozotocin- induced hyperglycemia in rats reduces mitochondrial dysfunction, improves the myocardial energy state and reduces damage to cell membranes in the AAR of reperfused myocardium, thus reducing the MI size

The present study confirms the effect of STZ-induced DM on myocardial susceptibility to IRI in rats. We showed that G administration significantly reduces MI upon reperfusion restoration. This benefit is due to the induction of intracellular signaling through the G-protein-coupled transmembrane receptors GalR1, GalR2, and GalR3 (*Fig. 5*). The protective effect of G manifested itself in less mitochondrial dysfunction, resulting in an improved energy state of the reperfused myocardial region. These positive shifts in the myocardial energy state were accompanied by a reduction in damage to the cell membrane. Taken together, the obtained results indicate that it is possible to use G as an accompanying therapy in DM1 complicated by myocardial IRI. In this regard, further study into the molecular mechanisms that reduce reperfusion stress in the diabetic myocardium using native and modified galanin peptides seems an important direction.

## Abbreviations

ROS: reactive oxygen species
AEC: adenylate energy charge
RC: respiratory control
AAR: area at risk
MI: myocardial infarction
IRI: ischemia/reperfusion injury
CK MB: creatine kinase MB
LDH: lactate dehydrogenase
LV: left ventricle
mt-CK: mitochondrial creatine kinase
OP: oxidative phosphorylation
LAD: left anterior descending coronary artery
LPP: lipid peroxidation
DM: diabetes mellitus, STZ streptozotocin
CK: creatine kinase
Cr: creatine
G: galanin
PCr: phosphocreatine
ΣAN: total adenine nucleotide pool
ΣCr: total creatine
TTC: 2,3,5-triphenyltetrazolium chloride
